# The Role of Salvage in the Management of Patients with Sinonasal Squamous Cell Carcinoma

**DOI:** 10.3390/biomedicines10061266

**Published:** 2022-05-28

**Authors:** Urszula Kacorzyk, Tomasz Wojciech Rutkowski

**Affiliations:** Maria Sklodowska-Curie National Research Institute of Oncology Gliwice Branch, ul. Wybrzeże Armii Krajowej 15, 44-101 Gliwice, Poland; urszula.kacorzyk@io.gliwice.pl

**Keywords:** sinonasal squamous cell carcinoma, salvage

## Abstract

Objectives: This paper presents and discusses the rate and outcome of salvage according to various factors for patients with sinonasal squamous cell carcinoma (SNSCC). Methods: Data of 79 patients treated radically due to SNSCC between 2000 and 2016 in the National Cancer Research Institute, Gliwice branch, were analyzed. Surgery was the primary treatment in 63 (79%) of patients. The ratio, type, and effectiveness of salvage was assessed and correlated with prognostic factors. Probabilities of overall survival (OS), local control (LC), nodal control (NC), and locoregional control (LRC) were assessed and compared between the groups. Results: The 5-year LC, NC, and LRC survival rates were 62%, 75%, and 53%, respectively. The 5-year OS rate was 51%. In 34 (43%) patients, treatment failure was reported, and salvage was performed in 17 (50%) of them. It was shown that patients after any salvage had significantly longer 2- and 3-year OS rates when compared to patients with no salvage: 52% vs. 7% and 38% vs. 0%, respectively (*p* = 0.004). Two- and three-year OS rates for patients after effective and ineffective salvage were 83% vs. 33% and 83% vs. 11%, respectively (*p* = 0.02). For patients with effective salvage, OS did not differ significantly when compared to the OS of primarily cured patients (*p* = 0.6). Conclusions: For SNSCC patients after treatment failure, salvage is possible in half of the cases and can improve their overall survival even if not finally successful. Moreover, effective salvage can compensate for the failure and give the same ultimate OS as in primarily cured patients.

## 1. Introduction

Completed surgical resection followed by postoperative radiotherapy (RT), alone or combined with chemotherapy (CHT), is considered the best treatment option [1,2,3,4,5] for patients with sinonasal malignant tumors (SNM). Local recurrence remains the main reason of treatment failure and is diagnosed in about 50% of those patients [6,7,8,9]. In such cases, salvage therapy should be considered. As the term salvage therapy is not well defined and may also concern a final attempt, it should be stressed that the context used in this manuscript is a second attempt of radical treatment. The question arises as to how effective the salvage procedure offered to these patients actually is. Data on the results of salvage options for patients who failed primary treatment due to SNM remain sparse, mostly due to the rarity of these tumors. Histopathological diversity also makes it difficult to draw conclusions about the role of salvage in general. Squamous cell carcinoma is the most frequent histopathological type of malignancy arising from the sinonasal region. This paper presents and discusses the rate of salvage and its outcome according to salvage type, time, and other selected factors in patients after radical treatment for SNSCC.

## 2. Material and Methods

A review of retrospective clinical data of 233 consecutive patients with either nasal cavity or paranasal sinus tumors treated between 2000 and 2016 in the National Cancer Research Institute, Gliwice branch, was performed. The study was conducted according to the guidelines of the Declaration of Helsinki and approved by the Ethics Committee of Maria Sklodowska-Curie National Research Institute of Oncology, Gliwice Branch (decision code: KB/430-73/21 date of approval: 10 May 2021).

Persistent disease was defined as either a local or regional tumor that did not disappear after treatment or recurred within 6 months of treatment completion. Recurrence was defined as either a local or regional tumor that recurred later than 6 months of treatment completion, or that recurred anytime in patients who underwent postoperative treatment.

Both cumulative survival and tumor control rates were calculated using the Kaplan–Meier product-limited (actuarial) method. A *p* value of <0.05 was considered statistically significant. Detailed analysis of the time and site of the primary treatment failure was performed. The ratio and effects of salvage were analyzed. The distributions of the discrete variables in various groups of patients were compared by means of Fisher’s exact test. As of the end of RT, the Kaplan–Meier product-limit estimate was used to estimate the probabilities of overall survival (OS), local control (LC), nodal control (NC), and locoregional control (LRC), defined as primary tumor control, regional nodal control, and LC and/or NC, respectively. The log-rank test was adopted to make a comparative analysis.

Salvage treatment was defined as an attempt to apply the radical management of persistent tumor or recurrence after the completion of primary radical therapy. Successful (effective) salvage was reported when the treated tumor was either no longer observed for at least 3 months or remained stable for at least 6 months after the salvage procedure. Subsequent recurrence was defined as either a recurrence or progression following the previous salvage.

Analysis of the treatment outcome was based on follow-up data. Patients were seen 1 to 2 months after treatment completion, then every 3 months for the first year, every 6 months for another year, and then annually. At each follow-up visit, a physical examination, including the palpation of the neck, was performed. Routine imaging was achieved with MRI, CT, or positron emission tomographic scans every 6 months, or at the physician’s discretion based on physical examination findings.

## 3. Results

### 3.1. Patients Characteristcs

As many as 74 patients undergoing a palliative approach and 12 patients with benign tumors were excluded from the study. The remaining group of 146 patients consisted of subjects with squamous cell carcinoma (SCC), diagnosed in 79 (54%) cases, followed by adenoid cystic carcinoma and with undifferentiated sinonasal cancer, diagnosed in 21 (14%) and 20 (13%) cases, respectively. Due to the variety of clinical scenarios and different management approaches according to various histopatological types, further analysis was carried out only in the group of 79 SCC patients. The group consisted of 50 (63%) males and 29 (37%) females with a median age of 58 years. Additionally, 43 patients (54%) had never smoked and 36 (46%) were smokers. The median duration of symptoms before diagnosis was 4 months. The maxilla was the primary tumor localization in 51 (64.5%) cases followed by the nasal cavity in 22 (28%) cases. Tumor stage (T) was assessed retrospectively according to the 8th edition of TNM classification and was locally advanced (T3 or T4) in 51 (64.5%) patients. Primary nodal involvement (N) was found in only 16 (20%) cases.

The characteristics of the patients, tumor type, and treatment are presented in Table 1. All patients underwent radical treatment. Surgery was the primary treatment approach in 63 (79%) patients and followed induction CHT in 6 patients. No surgical treatment was provided to 10 patients. In this group, radiochemotherapy (CHRT) or RT alone was administered to two and three patients, respectively, and induction CHT followed by CHRT was administered to five patients. All CHT sessions were platinum-based. Monochemotherapy was used as concomitant therapy during RT. Platinum combined with either 5-FU as PF or taxanes as TPF was used as induction.

### 3.2. General Outcome of the Primary Treatment

**Figure 1:**
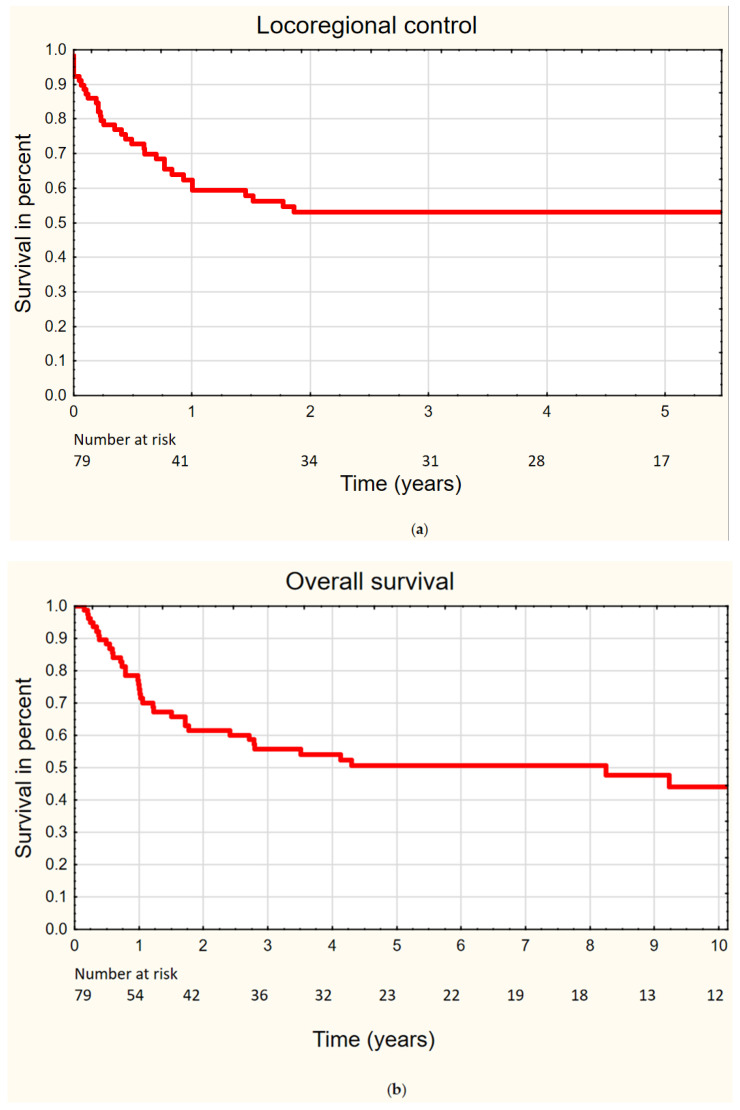
(**a**) Locoregional disease-free survival, (**b**) overall survival.

The median follow-up was 34 months. Locoregional treatment failure was found in 34 (43%) patients. The trend for higher ratio of locoregional failure was reported for patients who underwent primary treatment without surgery (*p* = 0.07). No significant relationship was found between the ratio of treatment failure and primary tumor localization, age, sex, T, smoking, or symptom duration (Table 1). Distant metastases were noted in two (9%) patients. It was found that the ratio of locoregional failure was significantly higher for patients with N+ (*p* = 0.02). Regarding the studied group, the 5-year local, regional, and locoregional disease-free survival rates were reported as 62%, 75%, and 53%, respectively (Figure 1a). The 5-year and 10-year OS rates amounted to 51% and 44%, respectively (Figure 1b).

### 3.3. Details of the Primary Treatment Failures

In 34 (43%) patients, treatment failure was observed during follow-up as a persistent (10/29%) or recurrent (24/71%) disease. Local recurrences (also when combined with nodal) were found as a result of physical examination (n = 9), surveillance CT (n = 4), or MRI (n = 3) in the absence of any physical findings. No data on recurrence diagnosis were reported in one case. Solitary regional failure was found based on a routine physical examination (n = 6) or surveillance CT (n = 1).

### 3.4. Salvage Treatment

Treatment failure significantly decreased the OS ratio. Local failure significantly decreased OS from 66% to 19% in the fifth year and from 55% to 19% in the tenth year of follow-up (*p* = 0.0001). Nodal failure decreased the 5- and 10-year OS rates from 57% to 23% and from 49% to 23%, respectively, (*p* = 0.03). Altogether, patients with locoregional failure had 5- and 10-year OS rates amounting to 20% and 20%, respectively, while those with locoregional control presented 72% and 59% 5- and 10-year OS rates, respectively (*p* = 0.00003).

The median time to primary treatment failure (including both persistent and recurrent disease) in the whole group was 3.5 months. For patients eligible for salvage, this median time was 7 months, and for those who were not eligible is was 1 month (*p* = 0.02). The median time to treatment failure after salvage for patients with effective salvage and non-effective salvage was similar, and equaled 5.5 and 7 months, respectively (*p* = 0.3).

### 3.5. Persistent Disease

Out of 10 patients with persistent local tumor, in two (20%) cases salvage treatment was undertaken. In one (50%) of these patients, this treatment was successful, which means that the patient was cured, with a survival of 167 months after salvage. In one (50%) patient, salvage failed, and the patient died 12 months later. With regard to patients with persistent disease, the median survival time was not significantly longer for subjects who underwent salvage (90 months, range: 12–167 months) than for those with no attempt at salvage (6 months, range: 2–15 months), *p* = 0.08.

### 3.6. Recurrent Disease

Out of 24 (30%) patients with tumor recurrence, salvage was undertaken in 15 (62.5%) cases. Among 10 patients with isolated local recurrence, salvage was performed in five (50%) patients but was successful in only two cases. In one patient, RT alone as a salvage was used, and the patient was cured. In the second patient, after primary surgical salvage, three subsequent recurrences were noted. All of them were resected, followed by RT to achieve durable cure. Out of seven patients with solitary nodal recurrence, salvage was also performed in five cases (71%) and was successful in three (60%) patients. In all these three patients, surgery was performed, followed by RT in one case. No subsequent nodal recurrences were found in these patients. Out of seven patients with both local relapse and cervical adenopathy, salvage was possible in five (71%) patients, however none benefited from the procedure. Altogether, for patients with recurrent disease, salvage was successful in five (33%) subjects, giving an additional median time of 118.5 months of survival (range: 4–172 months).

### 3.7. Results of Salvage in the Entire Group

Overall, out of 34 patients with primary treatment failure, salvage was performed in 17 (50%) patients. Significantly more patients underwent salvage after recurrence (62.5%) than due to persistent disease (20%), *p* = 0.02. In 12 (70%) patients, monotherapy was administered, and in 5 (30%) patients, a combined salvage option was applied. In seven patients, surgery alone was the primary choice of salvage, giving a permanent cure in three (43%) cases. RT was the only salvage procedure in four patients, with one case being cured (25%). Chemotherapy alone was given to one patient but without effect. In four cases, combined salvage option was performed due to subsequent recurrences, trying to “catch the escaping disease” and in one case salvage surgery was supplemented with RT directly, resulting in cure. In another patient, three subsequent recurrences were resected and the fourth recurrence was irradiated, which also resulted in cure. The other three patients failed their combined salvage. Details about the effectiveness of salvage option according to the type and site of failure, including subsequent failures, are presented in Table 2.

**Figure 2:**
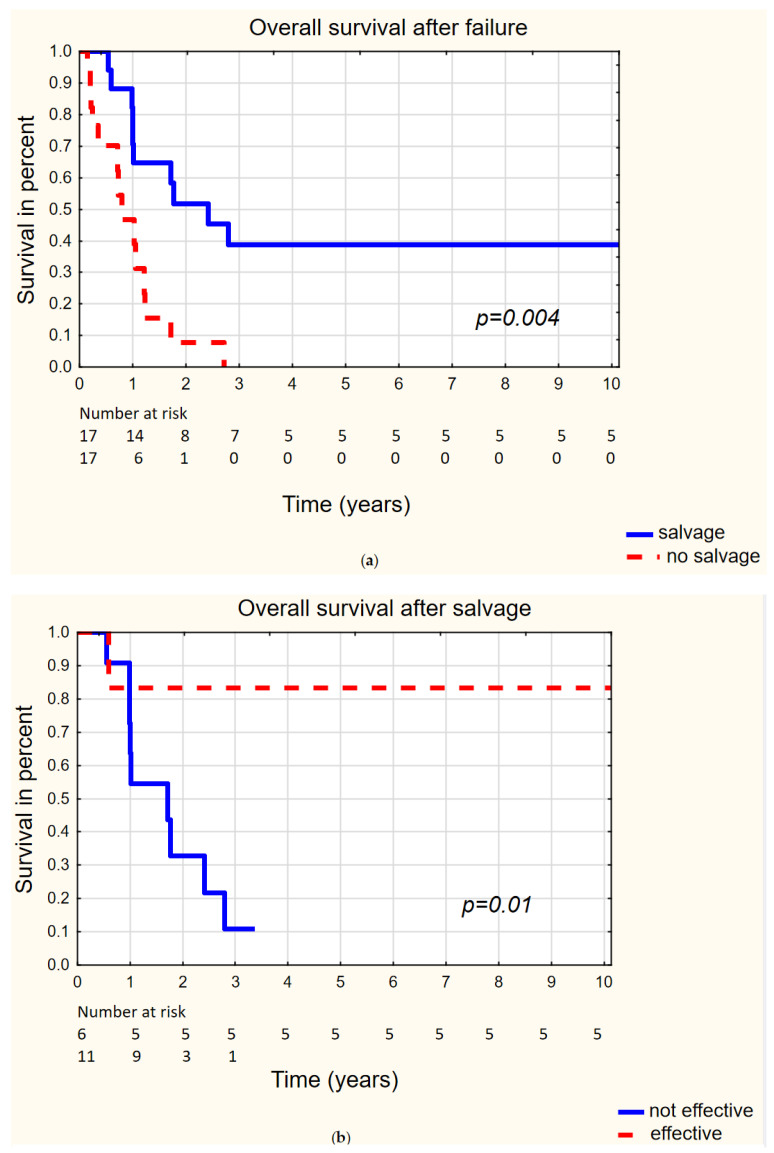

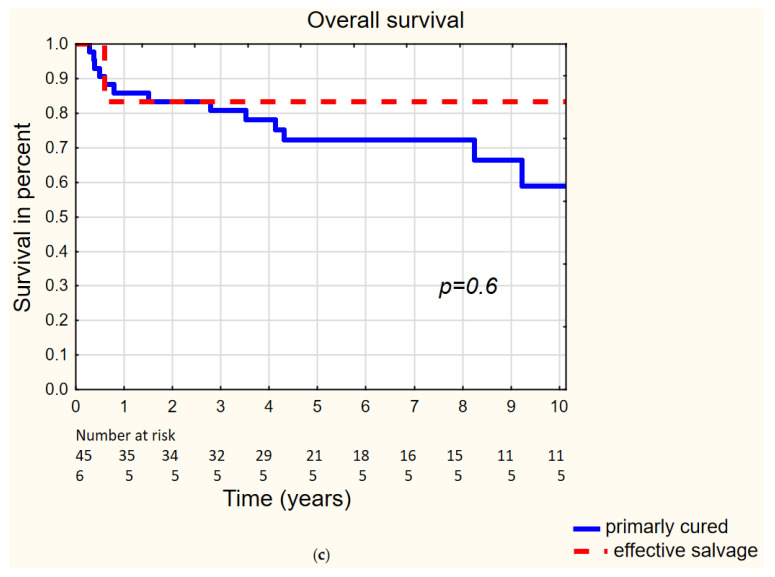
Overall survival considering salvage after primary treatment failure. (**a**) Patients with treatment failure: salvage vs. no salvage. (**b**) Patients after salvage: effective salvage vs. ineffective salvage. (**c**) Patients cured primarily vs. cured after salvage.

The salvage attempt, with no regard of its effectiveness, gave significantly longer additional median time of survival among patients qualified for salvage (8 months) than among those disqualified for salvage (2 months) *p* = 0.007. OS turned out to be significantly longer for those after any salvage versus those with no attempt at salvage. Two- and three-year OS rates for patients after salvage and with no salvage were 52% vs. 7% and 38% vs. 0%, respectively (*p* = 0.004) (Figure 2a). Effective salvage gave the median time of additional survival of 118.5 months, which was significantly longer than the time given with no effective salvage (10 months) (*p* = 0.04). This led to significantly longer OS after effective salvage vs. ineffective procedure. Two- and three-year OS rates for patients with effective and ineffective salvage were 83% vs. 33% and 83% vs. 11%, respectively (*p* = 0.02) (Figure 2b). Consequently, for patients with effective salvage, OS did not differ significantly when compared to the OS of primarily cured patients (*p* = 0.6) (Figure 2c). Due to the results of salvage, ultimately, the 5- and 10-year LC increased from 62% to 66%, NC increased from 75% to 79%, and LRC increased from 53% to 61%.

### 3.8. Salvage Surgery

Surgery was the most frequent type of salvage, both as a single approach (7/54%) and as a component of combined salvage treatment (4/80%) (Table 2), being the first salvage approach in all 11 patients. Out of 11 patients in whom salvage surgery was performed, a tumorectomy, extended resection of the middle face, radical lymphadenectomy, or both extended resection and lymphadenectomy were applied in 2 (18%), 3 (27%), 3 (27%), and 2 (18%) cases, respectively. In one case, no details about the salvage surgery approach were given.

### 3.9. Salvage Radiotherapy

RT as a salvage approach was utilized in 9 (53%) out of 17 patients. In such cases, the dose of RT and the technique was adjusted to the individual clinical situation, considering the localization of the tumor, critical normal organs, and previous therapies. RT as a salvage monotherapy was carried out in four (23.5%) patients. In three cases, 20Gy in five fractions was given to the Gross Tumor Volume (GTV); in one case this was 66 Gy, as a simultaneous integrated boost given to GTV.

## 4. Discussion

The best treatment option for patients with SNSCC is a multimodality approach, including both surgery and RT [1,2,3,4,5]. Locoregional failure is observed in about 50% of all cases with the predominance of local recurrence with the 5-year local control rates of 49–62% [6,7,8,9] for surgical treatment combined with adjuvant therapy, which may decrease to 20% [10] for definitive RT or CHRT alone.

Although the post-treatment surveillance seems to be essential for early detection of failure, there is still little evidence to suggest that early detection of recurrences adds significant benefit to survival. Considering, however, that the majority of failures appear within 2 years, a close follow-up is more important during the first 24 months to give a better chance of a cure, especially in terms of curative salvage treatment. De Felice et al. suggest clinical exam with fiberoptic examination every 3 months for 2 years and every 6 months thereafter and an imaging exam (CT or/and MRI) performed every 6 months up to the second year and then annually [11]. Regional relapse is diagnosed in a minority of patients with SNSCC (5–11%) [6,7,8,9]. The 5-year OS is in the range of 40–71% [6,7,9,12,13,14]. In our material, recurrence was found about 2.5 times more often than persistent tumor. Recurrent disease is also considered the reason for failure more often than persistent tumor in other reports [1,7,9]. In most cases, as with our patients, recurrence appears in the median time of 8–16 months [6,7,9]. It has been recommended that patients with local or locoregional recurrence should be discussed within a multidisciplinary team to consider the possibility of a salvage surgery or reirradiation which is a curative treatment strategy. However, this is possible only in selected cases [15].

Despite recommendations, not much is known about the ratio of salvage or who could be offered salvage as a “second chance” therapy in the case of recurrence in this group of patients. It is also difficult to predict how effective it could be for patients who have experienced failure. It is even more challenging due to histopathological diversity, making it difficult to conclude which salvage would be optimal in general. As SCC is the most frequent histopathological type of malignancy arising from the sinonasal region, our analysis concentrated on this subpopulation of patients. Our results showed that even 50% of patients suffering from SNSCC, who failed after primary treatment, may undergo salvage procedures. Michel et al. described the results of treatment in 33 SNSCC patients and reported salvage in 71% of the cases but did not describe its outcome [1]. Hoppe et al. described local or regional relapses in 37 (44%) out of 86 patients, but SNSCC was diagnosed in only 49% of them. In addition, 30% of patients with failure underwent a radical salvage attempt (surgery or/and RT). An additional 8 to 65 months of life was reported after this salvage, whereas patients who underwent only supportive care died after just 4 months [6]. In our group, surgery was reported to be the most effective—although not significantly—and most frequently performed salvage procedure. Birgi et al. described eight patients with local recurrence, involving one in combination with regional failure, in a group of 43 SNSCC patients. Two patients (25%) with an early stage disease underwent successful surgical salvage of local recurrence, but the remaining six patients were managed with palliative approaches. Four patients who experienced regional lymph node recurrence included one with simultaneous local recurrence. Altogether, salvage was performed in 33% of the patients with failure in this group of subjects [5]. Dirix et al. reported 38% of the patients with SCC in the group of 127 subjects. Regardless of pathology, local failure was found in 42.5% of the cases, but no salvage approach for them was reported in this series. Contrary to this, in our group of patients, salvage was possible in 50% of all local recurrences. In the case of nodal recurrence, salvage surgery followed by postoperative RT was possible in all 6 cases (100%) in Dirix et al. group. In our series, it was possible in about 70% of regional or both regional and nodal failures. Three patients were free from disease for 2, 5, and 13 years after the regional recurrence, respectively. Another three patients ultimately died from their disease because they subsequently developed distant metastases [7]. Li et al. described the results of treatment in 107 patients with primary SNSCC treated between 1996-2007. They found recurrence in 44 (41%) patients, and 33 (75%) cases underwent salvage surgery; the 5-year survival rate after salvage surgery was 29.1%. Due to the fact that the article is in Chinese, not many details could be obtained from this study [16].

The surveillance of our patients showed that the time to failure may be of predictive value, indicating those who may benefit more from salvage. We found the longer the time from treatment completion to treatment failure, the higher the chance for salvage. According to our findings, those who underwent salvage, irrespective of the final result, had significantly longer OS than those who were not eligible. As for other types of head and neck cancer, surgery should be considered the main radical salvage option for patients with primary treatment failure, although we were not able to confirm its significant advantage in our series, probably due to the low number of cases. Moreover, effective salvage compensated for the failure and gave the same ultimate OS as primarily cured patients.

For patients with oral cavity cancer, recurrence interval was found to be a strong prognostic factor, and patients with less than 18 months disease-free interval tended to have a less favorable outcome [17]. Finally, it should be stressed that, after primary salvage, subsequent salvage approaches are possible, and the chance of success still exists.

This study has several limitations. Some of them are related to the retrospective nature of collected data, which may not be precise enough in some respects such as type of primary surgical approach or details of postsurgical pathological findings. Other constraints are linked to the rarity of these tumors and generally low number of analyzed cases. The results of this dilemma have been found in a restricted number of publications, and there is an almost total lack of results of randomized clinical trials dedicated to patients with sinonasal malignancies.

## 5. Conclusions

In summary, it could be said that patients after radical treatment due to SNSCC should remain under oncological surveillance, as salvage therapy is possible in half of those with failure and can improve their overall survival even if not finally successful. Moreover, effective salvage can compensate for the failure and give the same ultimate OS as in primarily cured patients.

## Figures and Tables

**Table 1:** The clinical characteristics of patients and the results of primary and salvage treatment according to selected clinical parameters. LRf—locoregional failure, Lf—local failure, Rf—regional failure, Sim—simultaneously, RT—radiotherapy, CHT—chemotherapy, and CHRT—radiochemotherapy.

	No (%)	Primarily Cured (%)	Treatment Failure					Salvage/Effective	*p*
			LRf (%)	*p*	Lf	Rf	Sim Lf and Rf		
Maxillary sinus	51 (64.5)	27 (53)	24 (47)	0.3	13	5	6	9/3	0.9
Nasal cavity	22 (28)	14 (63.5)	8 (35)		4	2	2	6/2	
Other	6 (7.5)	4 (66.5)	2 (33.5)		1	0	1	1/2	
Age (years)				0.9					0.1
≤58	40	24	16		9	4	3	7/1	
>58	39	21	18		9	3	6	10/5	
Male	50 (63)	26 (52)	24 (48)	0.9	14	4	6	12/4	0.4
Female	29 (37)	19 (65.5)	10 (34.5)		4	3	3	5/2	
T1	5 (6)	2 (33)	3 (67)	0.9	3	0	0	3/0	0.9
T2	23 (29)	16 (69)	7 (31)		3	2	2	5/2	
T3	17 (21.5)	6 (35)	11 (65)		7	2	2	5/2	
T4	34 (43)	21 (61)	13 (39)		5	3	5	4/2	
N+	16 (20)	5 (31)	11 (69)	0.02	3	3	5	4/2	0.9
N0	63 (80)	40 (63)	23 (37)		15	4	4	13/4	
Smokers	43 (54)	26 (60)	15 (40)	0.1	8	3	4	5/1	0.3
Non-smokers	36 (46)	17 (47)	19 (53)		10	4	5	12/5	
Duration of symptoms (months)				0.4					0.3
≤4	41 (52)	23 (56)	18 (44)		9	4	5	12/5	
>4	38 (48)	22 (58)	16 (42)		9	3	4	5/1	
Surgery followed by RT/CHRT	63 (79)	39 (62)	24 (38)	0.07	11	9	7	14/5	0.9
RT/CHRT	5 (7)	1 (20)	4 (80)		1	0	0	2/1	
Induction CHT	11 (14)	5 (45)	6 (55)		3	0	3	1/0	

**Table 2:** Ratio of salvage and its effectiveness according to the category of primary treatment failure and the type of salvage. PD—persistent disease, R—recurrence, Lf—local failure, Rf—regional failure, LRf—local and regional failure, SR—subsequent recurrence, S—surgery, RT—radiotherapy, and CHT—chemiotherapy.

	Salvage/Success						
	PD	R	Lf	Rf	LRf	SR	Sum (Success in %)
Monotherapy							12/4 (33)
S	2/1	5/2	3/1	2/2	2/0	0	7/3 (43)
RT	0	4/1	2/1	1/0	1/0	0	4/1 (25)
CHT	0	1/0	0/0	0	1/0	0	1/0 (0)
Combined treatment							5/2 (40)
S + RT	0	4/2	2/1	2/1	0	2	4/2 (50)
RT + CHT	0	1/0	0	0	1/0	1	1/0 (0)

## Data Availability

Data supporting reported results can be obtained from corresponding author.
